# Preparation and Antigenic Site Identification of Monoclonal Antibodies against PB1 Protein of H9N2 Subtype AIV

**DOI:** 10.3390/vetsci11090412

**Published:** 2024-09-05

**Authors:** Yiqin Cai, Guihu Yin, Jianing Hu, Ye Liu, Xiangyu Huang, Zichen Gao, Xinyu Guo, Ting Jiang, Haifeng Sun, Xiuli Feng

**Affiliations:** 1 Key Laboratory of Animal Microbiology of China’s Ministry of Agriculture, College of Veterinary Medicine, Nanjing Agricultural University, Nanjing 210095, China; 2021107050@stu.njau.edu.cn (Y.C.); yinguihu@stu.njau.edu.cn (G.Y.); 2021207047@stu.njau.edu.cn (J.H.); 2023007051@stu.njau.edu.cn (Y.L.); 2020107044@stu.njau.edu.cn (X.H.); 2022107058@stu.njau.edu.cn (Z.G.); 2022807117@stu.njau.edu.cn (X.G.); jiangting@njau.edu.cn (T.J.); shf@njau.edu.cn (H.S.); 2 MOE Joint International Research Laboratory of Animal Health and Food Safety, College of Veterinary Medicine, Nanjing Agricultural University, Nanjing 210095, China

**Keywords:** H9N2 subtype AIV, PB1 protein, monoclonal antibody, antigenic site

## Abstract

**Simple Summary:**

H9N2 subtype avian influenza virus (AIV), which is mild but widespread, has been a concern due to the potential threat to poultry farming and public safety. AIV-infected poultry exhibit a complex range of various types of disease, which, coupled with the fact that AIV viruses are prone to genetic mutation and remodeling and exhibit a very high degree of variability, further increases the difficulty of immunization and diagnosis of avian influenza. We focused on the PB1 protein, a vital part of the virus’s ability to replicate. In this study, a truncated PB1 protein was designed and expressed in the prokaryotic expression system. Four hybridoma cells secreting the antibody specific to PB1 protein were screened using mouse immunization and cell fusion techniques. Two B cell antigenic determinants were identified by gradually truncating protein expression, and were conserved across different flu strains and located on the surface of PB1 protein. These findings could help make better vaccines against the H9N2 virus and might be important for controlling the spread of bird flu and protecting both animals and humans.

**Abstract:**

Recently, low pathogenic avian influenza virus (LPAIV), including H9N2 subtype, has been common clinical epidemic strains, and is widely distributed globally. The PB1 protein is a key component of the viral RNA polymerase complex (vRNP), and is vital to viral transcription and translation. In this study, to investigate the antigenic determinants in the PB1 protein, the truncated PB1 sequence (1bp-735bp) from H9N2 subtype AIV was amplified with PCR, and expressed in plasmid pET-28a (+). After purification, the recombinant PB1 protein was used to immunize BALB/c mice. Following immunization, hybridoma cells producing PB1-specific monoclonal antibodies were generated through the fusion of splenic lymphocytes with SP2/0 cells. Then, four stable hybridoma cell lines (5F12, 5B3, 2H9, and 3E6) were screened using indirect ELISA and Western blotting. Furthermore, two antigenic sites, 67NPIDGPLPED76 and 97ESHPGIFENS106, were identified through the construction of truncated overlapping fragments of the PB1 protein. These sites were conserved among 28 AIV strains, and were located on the PB1 protein surface. The findings offer a theoretical reference for the development and improvement of H9N2 vaccines and offer biological materials for virus detection during AIV infection mechanisms.

## 1. Introduction

Avian influenza, often referred to as AI, is a severe and rapidly spreading disease which is mainly caused by avian influenza virus (AIV), and the latter belongs to the Orthomyxoviridae family [1,2]. In 1966, H9N2 subtype AIV was reported to have been isolated from turkey flocks in Wisconsin for the first time [3]. The low pathogenicity H9 subtype AIV was initially reported in China in 1994 [4]. Notably, the 1997 AI outbreak in Hong Kong led to substantial poultry mortality and marked the first instances of avian-to-human transmission [5], posing a grave threat both to the healthy development of the poultry industry and to public health and safety.

The AIV genome encodes various viral structural proteins, in which alkaline polymerase 1 (PB1), alkaline polymerase 2 (PB2), and acid polymerase (PA) together constitute the virus RNA-dependent RNA polymerase complex. This complex is responsible for catalyzing virus RNA synthesis and plays a vital function in virus replication and transcription [6]. Among these components, the functional domains of the PB1 protein encompass the polymerase active region situated in the middle, the N-terminal nuclear localization (NLS), and interaction domains with PB2 and PA subunits [7]. The NLS at the N-terminal of PB1 directs its nuclear localization, which is a crucial process during viral RNA replication and transcription. The RNA polymerase’s active region, consisting of finger and palm domains, is responsible for transcribing RNA templates into mRNA [8]. Additionally, the interaction between PB1 and the cellular importin α/β complex mediates the translocation of viral RNA polymerase into the nucleus [9].

Mutations of the PB1 protein significantly influence the adaptability and pathogenicity of the influenza virus [10]. It has been reported that specific amino acid positions in PB1, such as 66S, 198K, and 701M, can enhance the pathogenicity of avian influenza viruses [11,12]. Furthermore, the PB1 protein interacts with host immune regulatory proteins, including interactions with RIG-I (Retinoic Acid-Inducible Gene I) receptors, which modulate the type I interferon (IFN-I) pathway within host cells [13]. Additionally, viral PB1 protein can degrade the innate immunity of the crucial adapter protein MAVS through selective autophagy mediated by host NBR1, hindering innate immune pathway signaling and type I interferon production [14], thereby promoting influenza virus replication. Therefore, the PB1 protein plays a significant role in the viral infection process.

Recently, H9N2 subtype AIV has increasingly become a predominant strain among clinical epidemics. It is essential to further examine the key determinants within the PB1 protein of the H9N2 subtype. The monoclonal antibodies targeting the PB1 protein and the identification of B cell epitopes hold significant implications for AIV infection diagnosis, vaccine development, antiviral drug discovery, and immunotherapy. To probe the novel determinants of the PB1 protein, in this study, a highly antigenic region of PB1 was selected and cloned, and expressed using a prokaryotic expression system. Following immunization of mice and cell fusion, four monoclonal antibodies targeting the PB1 protein were successfully generated, and two B cell epitopes were identified. Moreover, the analysis of the conservation and structural characteristics of these B cell epitopes plays a crucial role in understanding the function and antigenic structure of the PB1 protein in AIV, which facilitates a deeper comprehension of the viral pathogenic mechanism and offers guidance for prevention and novel therapeutic strategies against avian influenza.

## 2. Materials and Methods

### 2.1. Virus and Cells

H9N2AIV (strain A/chicken/Shandong/LY1/2017), examined in this research, was collected and maintained by our laboratory as referenced [15]. It was amplified in the allantoic fluid of 9-day-old specific-pathogen-free (SPF) eggs.

For the cultivation of SP2/0 cells, we utilized RPMI-1640 medium (350-000-CL, Wisent Biotechnology, Nanjing, China) supplemented with 20% Fetal Bovine Serum (FBS, C04001-500, VivaCell, Shanghai, China), along with 1% penicillin and 100 µg/mL streptomycin. The cells were kept in an incubator at 37 °C and 5% CO_2_.

HeLa cells were grown in a mixture of RPMI-DMEM medium (same source as above) containing 10% FBS (FSP500, ExCell, Suzhou, China), and the same concentrations of antibiotics as used for the SP2/0 cells. These cells were also maintained at 37 °C and 5% CO_2_.

### 2.2. Gene Amplification and Recombinant Expression of PB1

This step was based on the PB1 amino acid sequence from GenBank (MH018675.1). The PB1 protein’s antigenic hydrophobicity was utilized to identify a target region spanning amino acids 1 to 245 with a high likelihood of harboring B cell epitopes. Specific primers designed using Snapgene are detailed in Table 1.

According to the TRIZOL reagent procedure, allantoic fluid infected with H9N2 AIV was collected, total viral RNAs were extracted, and reverse transcription was employed to generate cDNA templates for PCR amplification. PCR conditions started with 94 °C initial denaturation, for 5 min, followed by 35 cycles of 94 °C denaturation for 30 s, 56 °C annealing for 30 s, and 72 °C extension for 2 min. PCR products were digested using *Bam*H I and *Hin*d III restriction enzymes and ligated into the pET-28a(+) vector, followed by transformation into E.coli BL21(DE3) strain to express the PB1 recombinant protein. The soluble recombinant protein was obtained and confirmed by SDS-PAGE and Western blotting.

Purification of recombinant PB1 proteins were conducted via Nickel Affinity Chromatography. Imidazole elution buffer containing 50–500 Mm of different concentrations of imidazole (imidazole, 50 mM Tris, 0.05 M NaCl) was used to elute the target protein, and the 80–150 Mm concentrations of imidazole eluate was collected as immune target protein.

### 2.3. Mouse Immunization

Five female BALB/c mice aged 6~8 weeks were immunized via intraperitoneal injection with 100 μg purified PB1 protein and Freund’s complete adjuvant by full oscillation emulsification. Then, on the 14th and 28th days after the initial immunization, the mice were immunized with PB1 protein and Freund’s incomplete adjuvant. Seven days after the last immunization, the antibody titers of the mouse serum were assessed using an enzyme-linked immunosorbent assay (ELISA), with purified PB1 recombinant protein employed as the coated antigen. Mice exhibiting high antibody titers were intraperitoneally injected with 200 μg PB1 protein without adjuvant on the third day prior to cell fusion to boost immunity.

### 2.4. Cell Fusion and Monoclonal Antibody (mAb) Preparation

Splenocytes were isolated from the boosted mice, and then fused with SP2/0 cells under the presence of polyethylene glycol (PEG4000) solution (P7171, SIGMA, St. Louis, MO, USA) on the third day following booster immunization. The fused cells were cultured with 20% FBS, 100 mg/mL streptomycin, 100 U/mL penicillin, and 1% HAT medium. The supernatant from the fused cells was screened by ELISA. The selected hybridoma cell line was then injected into the BALB/c mice to generate the ascites antibody. The type and isotype of the monoclonal antibody was detected with a mouse mAb isotype identification kit (PK20003, Proteintech, Rosemont, IL, USA). Subsequently, the specificities of the mAbs were assessed using Western blot and indirect immunofluorescence (IFA) techniques.

To enhance the titer of mouse ascites, each mouse was sensitized by intraperitoneal injection of 500 μL Freund’s incomplete adjuvant for one week, followed by injection of 200 μL containing 10^5^ to 10^6^ hybridoma cells. Ascites samples were collected one week after immunization.

### 2.5. ELISA Detection

To establish an indirect ELISA diagnosis, the purified recombinant PB1 protein served as the coating antigen. A total of 2 μg/mL purified recombinant PB1 protein was coated in CBS buffer (0.2 mol/L Na_2_CO_3_, 0.2 mol/L NaHCO_3_, pH = 9.6) at 4 °C overnight. After washing, the plate was blocked with PBST containing 5% skim milk and washed by PBST three times. Subsequently, the supernatant of hybridoma cells was incubated with the coated PB1 protein at 37 °C for 1 h. After washing, 100 μL with a dilution ratio of 1:5000 of HRP-labeled goat anti-mouse IgG antibody was incubated at 37 °C for 45 min. Next, 100 μL of TMB color solution was added into the plates, and color development proceeded at 37 °C for 15 min, and then was halted with 50 μL KPL termination solution. Absorbance was checked at 450 nm using an enzyme labeling instrument.

Furthermore, the titer of collected mouse ascites was determined by ELISA, and the subtypes of monoclonal antibodies were identified using a subtype identification kit (PK20002, Proteintech, Rosemont, IL, USA). The ascites antibody was diluted with 1× PBST at a ratio of 1:100,000 (50 μL/well). Sheep anti-mouse IgA + IgM + IgG-HRP was incubated with screened mAbs in plate wells for 1 h, and washed three times. Finally, the color developer and termination buffer were added into each well, and the data were analyzed following absorbance detection with a microplate reader.

### 2.6. IFA Experiment

HeLa cells were infected with H9N2 subtype AIV (MOI = 0.1) at 37℃, 5% CO_2_ for 24 h. Normal HeLa cells that were not infected with the virus were set up as a negative control. After washing, the cells were fixed with 4% methanol for 10 min at room temperature, and treated with 0.1% Triton for 10 min. Then, cells were incubated with the screened mAbs at 4 °C overnight. Then, the cells were incubated with CoraLite594-conjugated goat anti-mouse IgG (H + L) fluorescent antibody (SA00013-3, Proteintech, Rosemont, IL, USA) for 45 min, followed by incubation with DAPI. Subsequently, the cells were observed by fluorescence microscopy (Axiovert A1, Carl Zeiss AG, Jena, Germany).

### 2.7. Immunoblotting Analysis

The collected protein samples were separated through SDS-PAGE electrophoresis. The gel containing the target protein samples was then stained in Coomassie Brilliant Blue solution for 1 h and decolorized with decolorization solution.

For immunoblotting analysis, the gel containing the target protein was transferred onto a PVDF membrane pre-soaked in methanol following SDS-PAGE. Then, the transferred PVDF membrane was immersed in PBST solution containing 5% skim milk for 2 h, and was incubated with the corresponding primary antibody overnight at 4 °C on a shaking table. Subsequently, HRP-labeled goat anti-mouse IgG (H + L) diluted at 1:5000 was incubated with PVDF membrane at 37 °C for 45 min. After washing with PBST three times, the results of exposure and color development were observed under a chemiluminescence imager.

### 2.8. Design of Truncated PB1 Gene and Identification of B Cell Epitope

To ascertain the B cell epitope region recognized by the monoclonal antibody, a recombinant truncated plasmid was constructed for identification purposes. The truncated PB1 gene was designed according to the prediction results for the PB1 protein epitope by the Protean 7.1.0 software antigenicity binding website (http://tools.iedb.org/bcell/ (accessed on 3 January 2024)). The designed truncated gene, PB1-1 and PB1-95, was cloned by PCR and subsequently inserted into the pET-28a(+) vector utilizing restriction endonuclease *Eco*R I and *Sal* I. The positive recombinant plasmid was then transformed into *E. coli* BL21 (DE3 strain) for expression. Monoclonal antibodies and His-labeled antibodies were used as primary antibodies for Western blotting identification. To determine the final epitope, the truncated gene PB1-67, PB1-17, PB1-27, PB1-37, PB1-47, and PB1-57 was designed, and a prokaryotic expression plasmid was constructed using the cloned PB1 gene fragment and the pEGX-4T-1 plasmid. The recombinant plasmid was expressed and identified by Western blotting using a monoclonal antibody and a GST-tagged antibody. Primers for the aforementioned truncated genes are listed in Table 1.

### 2.9. Bioinformatics

The homology of the epitopes of the four monoclonal antibodies was examined using MEGA-X 10.1.7 software to investigate the formation of B cell epitope homology of the PB1 protein among different subtypes of AIV. Additionally, the structure of the PB1 protein of H9N2 AIV was predicted using the Zhang Lab’s tool (https://zhanggroup.org/I-TASSER/ (accessed on 26 February 2024)). Pymol 2.6 software was utilized to analyze the spatial characteristics and biological functions of the PB1 3D model and B cell epitopes based on the predicted modeling results.

## 3. Results

### 3.1. The Recombinant Construction and Expression of the PB1 Protein

**Figure 1:**
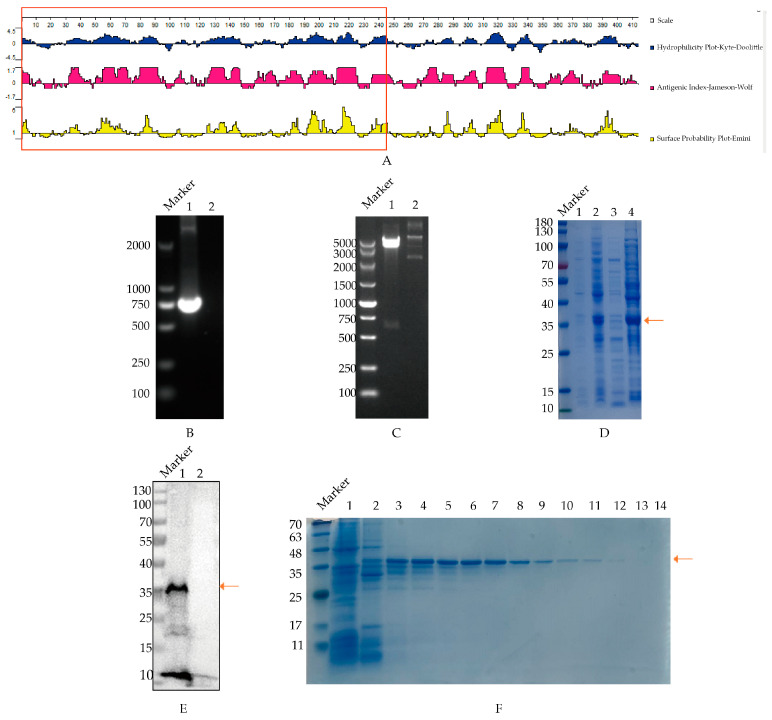
Expression and purification of truncated PB1 protein. The arrow in the figure refers to the position of PB1 protein. (**A**) Structural characteristics analysis of PB1 protein from H9N2 virus. The hydrophilicity, antigenic index and surface probability of the PB1 protein were analyzed with the Protean analysis software, where the designed fragment (1−245AA) was presented in the red-marked region. (**B**) PCR cloning of PB1 truncated gene. Canal 1, PB1 amplification, Canal 2, unrelated controls. (**C**) Determination of plasmid pET-28a-PB1 with double digestion. Canal 1, pET-28a-PB1 plasmid; Canal 2, pET-28a (+) vector control. (**D**) Identification of expressed PB1 protein. pET-28a-PB1 plasmid was transformed into BL21 strain, and expressed with IPTG induction, which was identified through SDS−PAGE analysis. Canal 1, the uninduced strain containing pET-28a-PB1 plasmid; Canal 2, the induced strain containing pET-28a-PB1 plasmid; Canal 3, supernatant of induced strain containing pET-28a-PB1 plasmid after sonication; Canal 4, precipitation of induced strain containing pET-28a-PB1 plasmid after sonication. (**E**) The reaction of recombinant PB1 containing His fusion protein with His antibody. Canal 1, sample of pET-28a-PB1 (36 KDa); Canal 2, control of host bacteria transfected with pET-28a (+) vector. (**F**) Isolation of the recombinant PB1 protein was carried out as follows: Canal 1 represents the initial flow-through post-column setup; Canals 2 through 14 correspond to the eluate post-elution utilizing varying imidazole concentrations. The sequence of imidazole concentrations used for elution was as follows: 50, 80, 80, 100, 100, 120, 120, 150, 150, 250, 250, 300, 500 mM, respectively.

The structural characteristics and antigenicity in the PB1 protein of H9N2 virus (A/chicken/Shandong/LY1/2017) using protein software are illustrated in Figure 1A. The results indicate that the 1-245AA region exhibited strong hydrophilicity and surface probability, suggesting promising antigenicity and B cell site potential for this region of the PB1 protein.

The truncated PB1 gene was successfully cloned through PCR amplification, which produced a 735 bp DNA fragment consistent with the expected size, as shown in Figure 1B. Subsequently, the cloned PB1 gene fragment was ligated into vector pET-28 (+) to be pET-28a-PB1, which was verified through double enzyme digestion using *Bam*H I and *Hind* III (Figure 1C). Next, the recombinant PB1 proteins were expressed following SDS-PAGE analysis, predominantly as inclusion bodies of approximately 36 kilodaltons (kDa), which was aligned with the anticipated size (Figure 1D). Furthermore, Western blotting demonstrated the specific reactivity of the expressed recombinant PB1 protein with the His tag antibody (Figure 1E), confirming good reactivity of the PB1 protein. Additionally, the recombinant PB1 protein was purified following Ni column affinity, with the protein eluted at an imidazole concentration of 80–150 mM, which was identified in SDS-PAGE analysis (Figure 1F). The original images of the Western blot are published as Supplementary Materials.

### 3.2. Screening of mAbs Specific to the PB1 Protein

**Figure 2:**
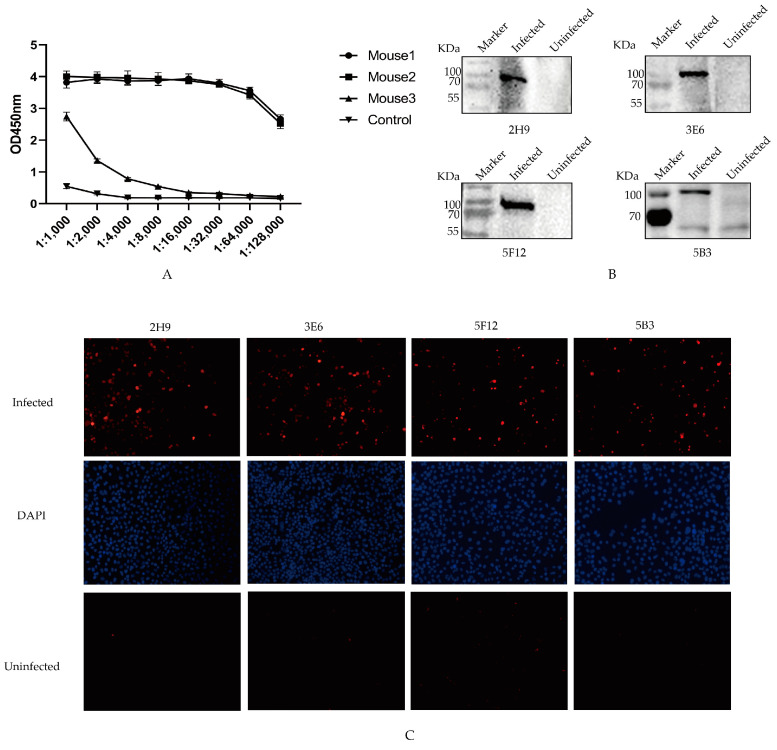
Specificity of four mAbs. (**A**) Antibody titers of three immunized mice. The sera were collected from the immunized mice on the seventh day after the third immunization, and antibody levels were measured. (**B**) Identification of mAbs. The protein samples from HeLa cells infected or uninfected with AIV interacted with 2H9, 3E6, 5B3, and 5F12 mAbs through Western blotting. (**C**) IFA was conducted to assess the interaction between the four mAbs and the Hela cells infected with AIV. These cells, post-infection with AIV, were exposed to the selected mAbs to evaluate their binding affinity to the PB1 protein component of AIV. The PB1 protein was marked in red; DAPI was marked in blue.

Antibody levels at 7th day after the third immunization were assessed via ELISA. Among all the mice, the highest antibody production was observed in Mouse 1 (Figure 2A). Consequently, mouse 1 was selected and boosted with 200 µg of recombinant PB1 protein to generate monoclonal antibodies.

After cell fusion and three rounds of subcloning, the cell supernatant was taken for ELISA detection, and four hybridoma cell lines that stably secreted anti-PB1 protein antibody were screened (2H9, 3E6, 5B3, and 5F12). The light chains of four mAbs subtypes targeting the PB1 protein were identified as Kappa chains using a mouse mAbs subtype detection kit. Additionally, the heavy chains of mAbs 2H9 and 3E6 were classified as IgG1 types, while those of 5B3 and 5F12 were classified as IgG2a types, as presented in Table 2.

To evaluate the specificity of mAbs in naturally recognizing the PB1 protein during viral infection, the protein samples of HeLa cells infected and uninfected with AIV were separately incubated with the four mAbs through Western blotting. The results showed the specific reaction band of native PB1 protein by the supernatants of four monoclonal cell strains (Figure 2B). Furthermore, immunofluorescence assay (IFA) results demonstrated intense red fluorescence in cells infected with H9N2 AIVs after utilizing mAbs as primary antibodies, whereas no fluorescence was observed in non-infected controls (Figure 2C). These findings suggest that all four screened mAbs might exhibit favorable reactivity toward the native conformation of the PB1 protein.

### 3.3. B Cell Epitope Identification of mAbs Target to PB1 Protein

**Figure 3:**
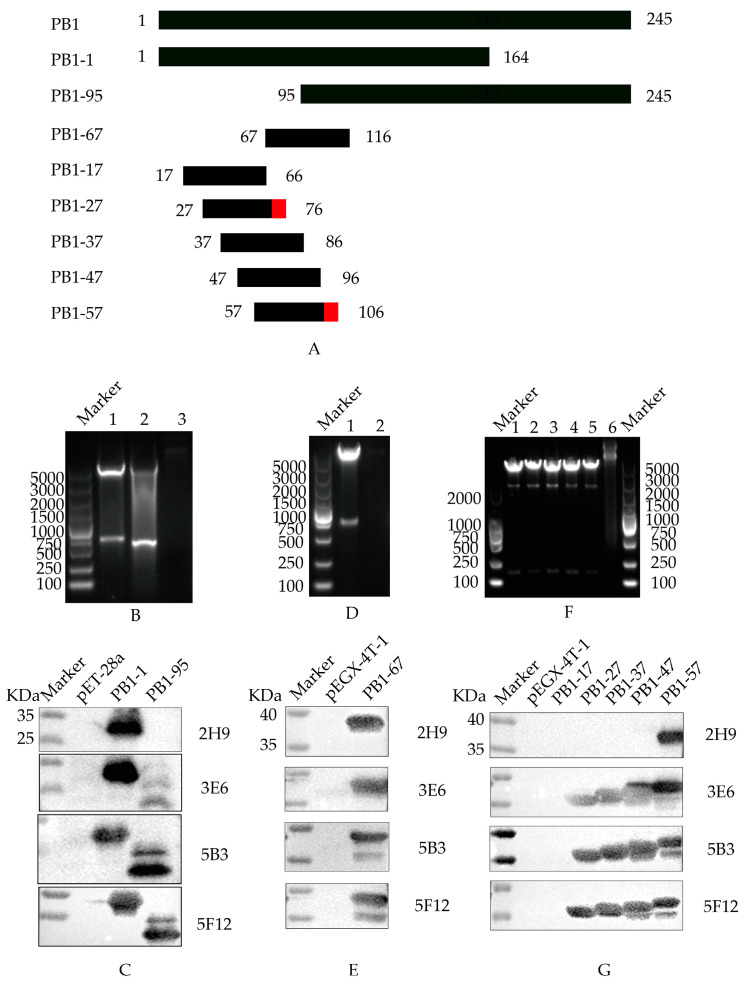
B cell epitope identification of PB1 mAbs. (**A**) Schematic diagram of PB1 protein. The red domain represents the amino acid region of the B cell epitope screened (**B**) Double digestion to identify the recombinant pET-28a-PB1-1 and pET-28a-PB1-95 plasmids. Canal 1: pET-28a-PB1-1; Canal 2: pET-28a-PB1-95; Canal 3: pET-28a empty load. (**C**) Western Blot identification of mAbs recognizing truncated PB1-1 and PB1-95 proteins. (**D**) Double digestion to identify the recombinant plasmid pEGX-4T-PB1-67. Canal 1: pEGX-4T-PB1-67; Canal 2: pEGX-4T-1 empty load. (**E**) Western Blot identification of mAbs recognizing truncated PB1-67 protein. (**F**) Double digestion to identify recombinant plasmids pEGX-4T-PB1-17, pEGX-4T-PB1-27, pEGX-4T-PB1-37, pEGX-4T-PB1-47, and pEGX-4T-PB1-57, respectively. Canal 1: pEGX-4T-PB1-17; Canal 2: pEGX-4T-PB1-27; Canal 3: pEGX-4T-PB1-37; Canal 4: pEGX-4T-PB1-47; Canal 5: pEGX-4T-PB1-57; Canal 6: pEGX-4T-1 empty load. (**G**) Western Blot identification of mAbs recognizing truncated PB1-17, PB1-27, PB1-37, PB1-47, and PB1-57 proteins, respectively.

To ascertain B cell epitopes recognized by the four selected monoclonal antibodies, a recombinant truncated PB1 protein was constructed and characterized (Figure 3A). Initially, the amino acid sequence of the PB1 protein was analyzed utilizing the Immune Epitope Database (IEDB) antigen tool. Following the principle of minimizing truncation of the antigen epitope, the PB1 gene was first truncated into two overlapping fragments, PB1-1 and PB1-95. These two genes were subsequently cloned into pET-28a(+) expression vector (Figure 3B) and analyzed by Western blot (Figure 3C). These results proved that 5B3, 3E6, and 5F12 mAbs exhibited reactivity with the PB1-1 and PB1-95 proteins, indicating that the recognized epitopes spanned amino acids 95 to 164. Conversely, 2H9 mAb exclusively reacted with the PB1-1 protein, suggesting that the recognized B cell epitopes targeting the screened mAbs spanned amino acids 1 to 94.

To further refine the B cell epitopes recognized by PB1 protein-specific mAbs, a PB1-67 protein truncation was designed and cloned into pEGX-4T-1 (Figure 3D). Following induced expression, the results of Western blot showed that 2H9, 5B3, 3E6, and 5F12 mAbs all demonstrated reactivity with the PB1-67 protein (Figure 3E), signifying that the epitopes recognized by mAbs 5B3, 3E6, and 5F12 spanned amino acids 95 to 116, while the B cell epitopes recognized by 2H9 mAb spanned amino acids 67 to 94.

To conclude the determination of the B cell epitope of the PB1 fragment, the amino acids 67 to 116 in the PB1 fragment were truncated into five overlapping peptides (PB1-17, PB1-27, PB1-37, PB1-47, and PB1-57). These sequences were cloned into pEGX-4T-1 (Figure 3F), followed by induced expression and Western blot analysis (Figure 3G). The 5B3, 3E6, and 5F12 mAbs exhibited reactivity with the PB1-17, PB1-27, PB1-37, PB1-47, and PB1-57 proteins, indicating recognition of the epitope-spanning amino acid ^67^NPIDGPLPED^76^. Conversely, the 2H9 mAb solely reacted with the PB1-57 protein, suggesting recognition of the B cell epitope-spanning amino acid ^97^ESHPGIFENS^106^.

### 3.4. Conservative Identification of B Cell Epitopes across AIV Strains

**Figure 4:**
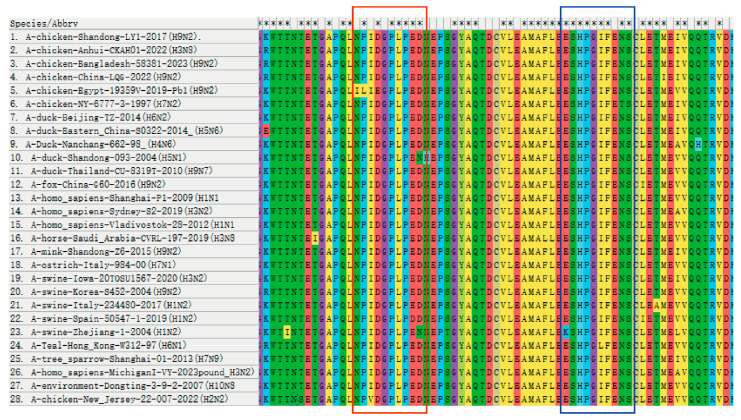
Conservative comparison of antigenic epitopes in the PB1 protein. Homology analysis of the identified determining sequences was performed in 28 strains by MEGA-X 10.1.7 software. The antigenic epitope ^67^NPIDGPLPED^76^ is marked in the red box and the antigenic epitope ^97^ESHPGIFENS^106^ is marked in the blue box. The “*” shown in the first line indicates that the amino acids are conserved in all strains of the virus, but the absence of “*” indicates that the amino acids are not conserved.

To investigate the conservation of the identified PB1 epitope, the range of 28 strains from different countries prevalent in recent years were compared. The results revealed that two B cell epitopes of the screened PB1 monoclonal antibody exhibited relative conservation across different subtype strains. Specifically, within the epitope ^67^NPIDGPLPED^76^, residues 67N, 68P, and 70D were only replaced by 67I, 68L, and 70E in A/chicken/Egypt/19359V/2019 (H9N2). Residue 69I was replaced by 69V in A/chicken/New Jersey/22 012428 007/2022 (H2N2). Residue 75E was replaced by 75D in A/swine/Zhejiang/1/2004 (H1N2), while 76D was replaced by 76N in A/duck/Shandong/093/2004 (H5N1) and A/swine/Zhejiang/1/2004 (H1N2). However, the remaining residues were conserved. Similarly, within the epitope ^97^ESHPGIFENS^106^, residue 97E was only replaced by 97K in A/swine/Zhejiang/1/2004 (H1N2), and residue 97E was only replaced by 102L in A/Teal/Hong Kong/W312/1997 (H6N1). All other strains exhibited conservation (Figure 4).

### 3.5. Spatial Structure Prediction of Epitopes in the PB1 Protein

**Figure 5:**
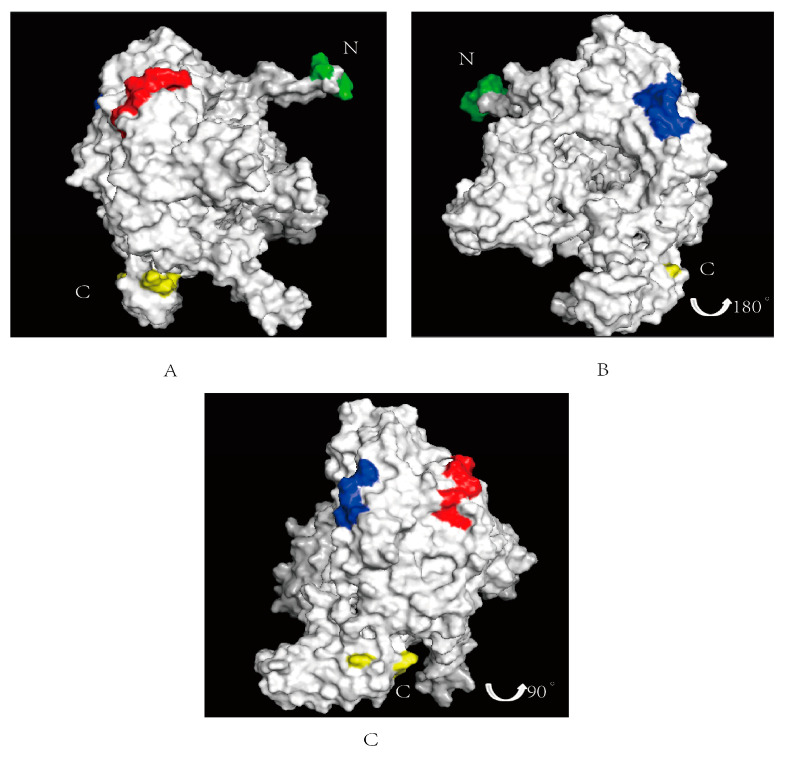
Spatial prediction of the PB1 protein with the identified two epitopes. The N-terminal of the PB1 protein is labeled in green, the C-terminal labeled in yellow. (**A**) The epitope identified ^67^NPIDGPLPED^76^ is marked in red on surface of the PB1 protein. (**B**) The epitopes identified ^97^ESHPGIFENS^106^ is marked in blue on the PB1 protein surface. (**C**) Integration of the two epitopes of the PB1 protein.

Based on ZhangLab (https://zhanggroup.org/ (accessed on 26 February 2024)), the spatial location of the identified epitopes in the PB1 protein were predicted by Pymol 2.6. The two epitopes are illustrated in Figure 5, with the epitope ^67^NPIDGPLPED^76^ labeled in red (Figure 5A) and the epitope ^97^ESHPGIFENS^106^ labeled in blue (Figure 5B). The N-terminal of the PB1 protein is labeled in green and the C-terminal labeled in yellow. The positions of these two epitopes on the PB1 protein are depicted in Figure 5C, revealing their surface localization within the PB1 protein. From the information available via Uniprot (https://www.uniprot.org/ (accessed on 14 March 2024)), we found that the epitope ^67^NPIDGPLPED^76^ was located in the Disordered region. The amino acid sequence in this region does not form a stable secondary structure, and it may be involved in the regulation of conductive molecules and other functions in the protein. Although ^97^ESHPGIFENS^106^ is not at the key binding site of the PB1 protein, Figure 5 shows that the epitope is located on the protein surface, and whether it binds to other receptors to play a role remains to be further explored.

## 4. Discussion

AIV infection poses a disastrous threat to the health of the poultry industry and human public safety. As a crucial component of core RNA polymerase in AIV, the PB1 protein plays a pivotal role during viral replication and regulation [16,17]. Screening viral protein epitopes with monoclonal antibodies is crucial for understanding the characteristics of viral proteins, identifying specific regions of the PB1 protein that elicit B cell immune responses, and revealing their importance in virus–host interactions.

In this study, we selected the region amino acids 1 to 245 in the PB1 protein of the H9N2 subtype AIV based on hydrophobicity and surface probability indices, which exhibited high antigenicity and B cell antigenic determinants. After recombinant truncated PB1 protein expression and mice immunization, four monoclonal antibodies (2H9, 3E6, 5B3, and 5F12) against the PB1 protein were screened based on cell fusion. Their reactivity with the viral PB1 protein was confirmed through various methods, including ELISA, Western blotting, and IFA. The results demonstrated that all four monoclonal antibodies could specifically recognize the PB1 protein.

Studies on B cell epitopes of the PB1 protein in AIV are limited. Some studies have identified potential B cell epitopes of the PB1 protein, providing insights into its immunogenicity and immune protection. The antibody epitopes in influenza internal proteins have been identified using serum from survivors of H5N1 infection, including the PB1 epitopes PB1 ^1348^MISKCRTKEGRRKT^1361^ and PB1 ^1420^TNGTSKIKMKWGMEMRRC^1437^ [18]. Also, a bioinformatics tool has been utilized to design peptides with high immunogenicity for immunization and screen B cell epitopes for the PB1 protein, including PB1 202–221, PB1 180–199, and PB1 458–477 [19]. In our study, two B cell epitopes, ^67^NPIDGPLPED^76^ and ^97^TITYSSPMMW^106^, were successfully identified by constructing truncated epitopes of screened monoclonal antibodies, which were the first reported PB1 protein determinant. Furthermore, through gradually truncating the PB1 protein, the recombinant plasmids containing PB1 17–66, 27–76, 37–86, 47–96, 57–106 were constructed and expressed, and analyzed with Western blotting. The results showed that the 2H9 monoclonal antibody could only recognize the sequence of 57–106. According to screening of the above amino acid sites, it was finally determined that the amino acid site that the 2H9 monoclonal antibody could recognize was ^97^ESHPGIFENS^106^, which was another screened epitope. These epitopes provide ideas for key targets in vaccine design and could be used to design vaccines that can stimulate the host immune system to produce an effective immune response. In addition, they can be used to identify and quantify specific pathogens or their components in immunodiagnostic reagents, which provides research ideas for the prevention and control of avian influenza.

Furthermore, these epitopes, identified through homology, were highly conserved across different strains and located on the surface of the PB1 protein. It is reported that the PB1 protein interacts with host immunomodulatory proteins in innate immunity, thereby affecting host natural immune signaling pathways and type I interferon production. The PB1 protein also affects host inflammatory signaling pathways, such as the NF-κB (Nuclear Factor-κB) pathway, which regulates inflammatory factor production [20]. These results might enhance the theoretical understanding of the PB1 protein antigen structure. However, the role of these epitopes in cellular immune responses and virus replication requires further investigation.

In summary, four monoclonal antibodies (2H9, 3E6, 5B3, and 5F12) against the AIV PB1 protein were screened in this study, and two B cell epitopes were identified. The key amino acids recognized by these antibodies were conserved among different AIV genotypes. The B cell epitopes identified are of great significance for vaccine research and immunodiagnostics. Localization of B cell epitopes is helpful for the design of new epitope vaccines with strong immunogenicity and high safety. Diagnostic combinations based on B cell epitopes can be established with multi-epitope peptides. The findings not only provide a tool for further exploration of the PB1 antigen structure and function, but also lay a necessary theoretical foundation for new AIV immunodiagnostic techniques.

## Figures and Tables

**Table 1:** Primers designed to target the PB1 gene for PCR and cloning.

Gene Name	Primer Name	Primer Sequence (5′–3′)	Fragment Length
PB1	PB1-F	CGGGATCCATGGATGTCAATCCGACT	735 bp
	PB1-R	CCAAGCTTCCCGGGTGTTGCGATTGC	
PB1-1	PB1-1-F	CCGGAATTCATGGATGTCAATCCGACTTTACTTT	492 bp
	PB1-1-R	ACGCGTCGACTATCAGCCTTCCTGATTCATTGGCT	
PB1-95	PB1-95-F	CCGGAATTCCTTGAAGAATCTCACCCAGGGATCT	453 bp
	PB1-95-R	ACGCGTCGACCCCGGGTGTTGCGATTGCCCTCCTC	
PB1-67	PB1-67-F	CCGGAATTCAATCCGATTGATGGACCACTACCTG	150 bp
	PB1-67-R	ACGCGTCGACTTGCTGAACAATTTCCATCGTTTCA	
PB1-17	PB1-17-F	CCGGAATTCGCCATAAGTACCACATTCCCTTATA	150 bp
	PB1-17-R	ACGCGTCGACGAGTTGGGGTGCTCCAGTCTCTGTG	
PB1-27	PB1-27-F	CCGGAATTCGACCCTCCATACAGCCATGGAACAG	150 bp
	PB1-27-R	ACGCGTCGACGTCCTCAGGTAGTGGTCCATCAATC	
PB1-37	PB1-37-F	CCGGAATTCGGATACACCATGGACACAGTCAACA	150 bp
	PB1-37-R	ACGCGTCGACATCTGTTTGTGCATACCCACTCGGC	
PB1-47	PB1-47-F	CCGGAATTCCATCAATACTCAGAAAAGGGAAAGT	150 bp
	PB1-47-R	ACGCGTCGACTTCAAGGAAAGCCATTGCTTCCAAT	
PB1-57	PB1-57-F	CCGGAATTCACGAACACAGAGACTGGAGCACCCC	150 bp
	PB1-57-R	ACGCGTCGACCGAGTTTTCAAAGATCCCTGGGTGA	

Note: The underline letters indicated the enzyme cleavage sites.

**Table 2:** Potency determination of PB1 monoclonal antibody.

mAbs	Mouse Ascites Potency	Heavy Chain	Light Chain
2H9	1:512,000	IgG1	Kappa
3E6	1:1,024,000	IgG1	Kappa
5B3	1:1,024,000	IgG2a	Kappa
5F12	1:1,024,000	IgG2a	Kappa

## Data Availability

The data presented in this study are available upon request from the corresponding author.

## Supplementary Materials

The following supporting information can be downloaded at: https://www.mdpi.com/article/10.3390/vetsci11090412/s1, The original images of the Western blot.
