# Supplementary material for: Preparation and Antigenic Site Identification of Monoclonal Antibodies against PB1 Protein of H9N2 Subtype AIV
Source: Vet Sci. 2024 Sep 5;11(9):412. doi: 10.3390/vetsci11090412 (PMC11435642; doi:10.3390/vetsci11090412)
Supplement: Supplementary file 1 [file vetsci-11-00412-s001.zip › vetsci-3086236-supplementary.pdf]

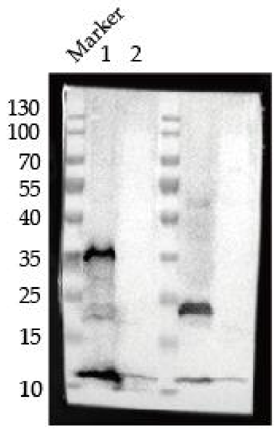

Figure 1E The recombinant protein PB1 containing His fusion protein was reacted with His antibody. Canal 1, sample of pET-28a-PB1 (36 KDa); Canal 2, pET-28a (+) vector control.

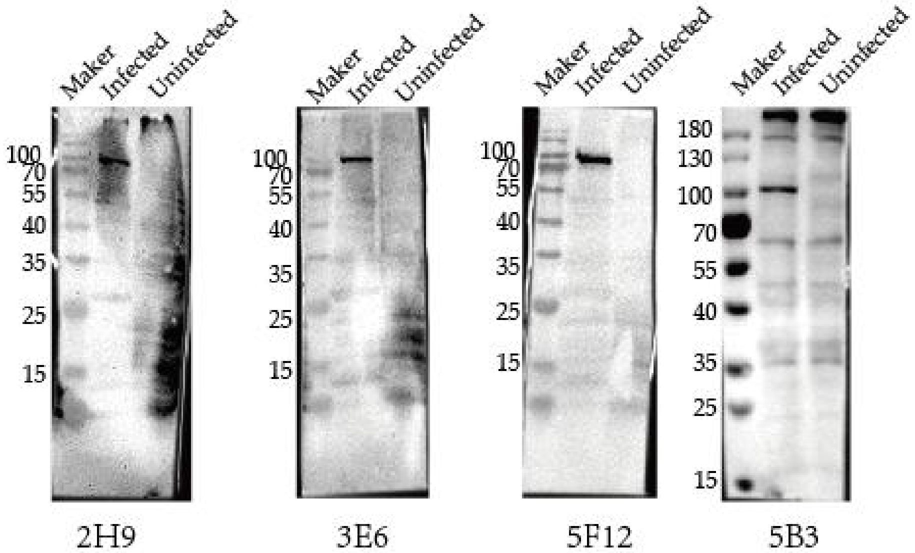

Figure 2B. The specific reaction between mAbs 2H9, 3E6, 5B3, 5F12 and PB2 protein from AIV-infected cells. Hela cells were infected with AIV, and the protein samples were collected to detect recognition between PB2 protein and mAbs by western blotting.

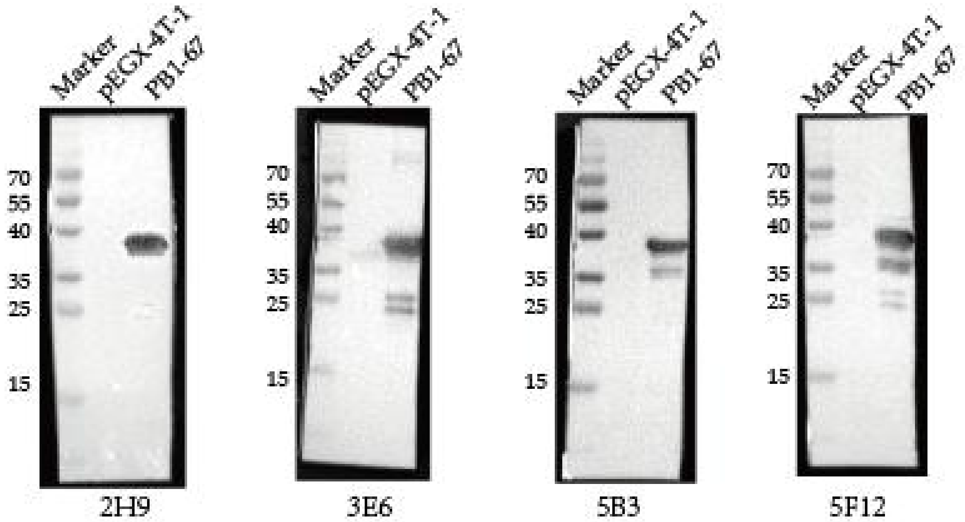

Figure 3C. Identification between truncated PB1-1 and PB1-95 with mAbs

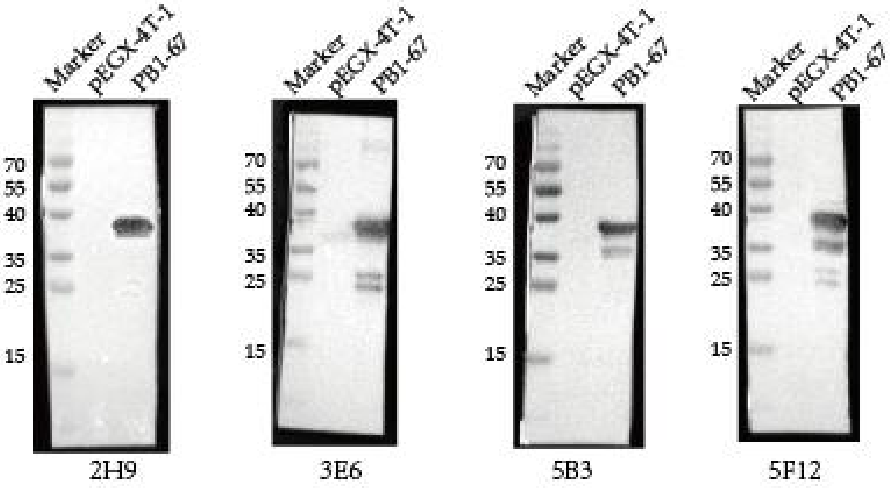

Figure 3E. Identification between truncated PB1-67 with mAbs

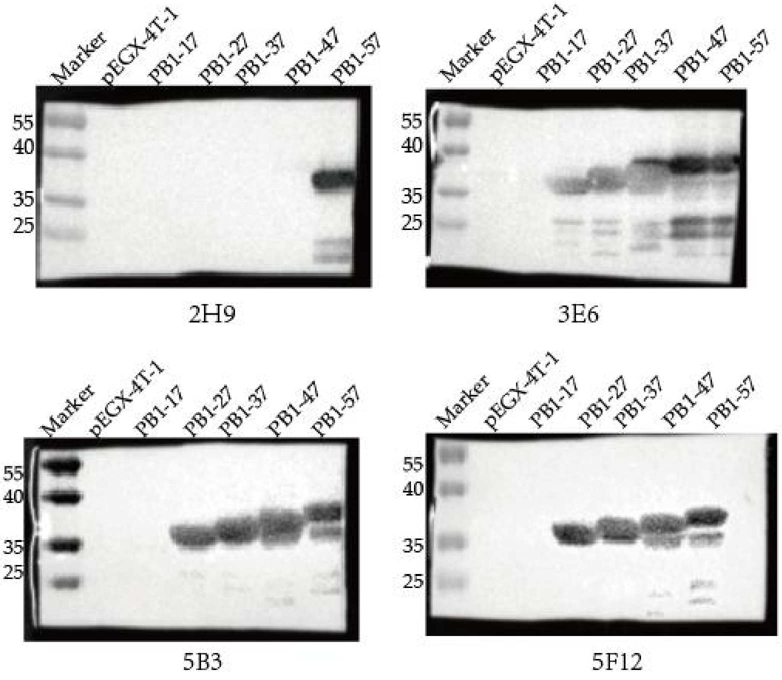

Figure 3G. Identification between truncated PB1-17, PB1-27, PB1-37, PB1-47, PB1-57 with mAbs
